# COVID‐19, thromboembolic risk, and Virchow's triad: Lesson from the past

**DOI:** 10.1002/clc.23460

**Published:** 2020-11-11

**Authors:** Jawahar L. Mehta, Giuseppe Calcaterra, Pier P. Bassareo

**Affiliations:** ^1^ Division of Cardiology University of Arkansas for Medical Sciences and the VA Medical Canter Little Rock Arkansas USA; ^2^ Postgraduate medical School University of Palermo Palermo Italy; ^3^ University College of Dublin Mater Misericordiae University Hospital Dublin Republic of Ireland

**Keywords:** blood stasis, COVID‐19, endothelial injury, hypercoagulability, SARS‐CoV‐2, thromboembolism

## Abstract

COronavirus Infectious Disease which started in 2019 (COVID‐19) usually presents with the signs and symptoms of pneumonia. However, a growing number of recent reports highlight the fact that the infection may be by far more than only a respiratory disease. There is evidence of an increased thromboembolic risk in COVID‐19 patients, with a variety of manifestations in terms of ischemic stroke, deep vein thrombosis, acute pulmonary embolism, acute myocardial infarction, systemic arterial embolism, and placental thrombosis. The German physician Rudolph Virchow, about two centuries ago, described three pivotal factors contributing together to thromboembolic risk: endothelial injury, hypercoagulability, and blood stasis. COVID‐19‐associated hypercoagulability is unique and distinctive, and has its own features involving the immune system. Many of the drugs proposed and currently undergoing evaluation for the treatment of COVID‐19 have one or more of the Virchow's triad elements as a target. The three factors outlined by Virchow are still able to explain the venous and arterial hypercoagulable state in the dramatic COVID‐19 setting. Nowadays, we have decidedly more sophisticated diagnostic tools than Virchow had, but many of the challenges that we are facing are the same as Virchow faced in the 19th century.

## 1 INTRODUCTION

Since its outbreak in December 2019, the world is facing a rapidly expanding pandemic owing to a new coronavirus, now named severe acute respiratory syndrome coronavirus‐2 (SARS‐CoV‐2). The SARS‐CoV‐2‐related disease (COVID‐19) usually presents with the signs and symptoms of pneumonia. However, several recent reports highlight the fact that the infection may be far more than only a respiratory disease, with the involvement of other organs.

The mechanism why some affected people suffer from disproportionate effect of the virus is still under debate. The patients present with or develop acute myocardial infarction, acute renal and liver injury, acute gastro‐intestinal issues, skin manifestations, neurologic damage, and other pathologies. Especially among older sicker patients, a worsening clinical presentation with acute respiratory distress syndrome and multi‐organ failure is seen quite often.^1^

## 2 COVID‐19 AND THROMBOEMBOLIC EVENTS

There is evidence of an increased risk of intravascular clot formation and disseminated intravascular coagulation (DIC) in patients with COVID‐19. Oxley et al were the first to report five cases of large‐vessel ischemic stroke in COVID‐19 patients aged less than 50 years, with a 7‐fold increase than what is normally expected. The patients had either no, or mild COVID‐19 symptoms.^2^ Similar reports came from the Netherlands. Investigators from that country found a remarkably high 31% rate of thrombotic complications, in terms of ischemic stroke, deep vein thrombosis, acute pulmonary embolism, myocardial infarction, systemic arterial embolism, and placental thrombosis, among 184 critical care patients with COVID‐19 pneumonia.^3^ After that, a number of reports from around the world provided confirmation of an increased thromboembolic risk in COVID‐19 patients, although with different estimates due to the different settings where the related data were gathered, that is, outpatient departments, hospital wards, or intensive care units, which influenced also the type of screening and diagnostic tests that were performed.^4^ Nevertheless, the rates of all thromboembolic events appear to be quite high among patients with COVID‐19, most of all in the most severe cases requiring admission to intensive care unit.^5^, ^6^, ^7^

When focusing on each specific thrombotic event, the rate of adverse venous thromboembolic events seems to be much higher in patients with COVID‐19 compared to those with non‐COVID‐19 infection and with acute respiratory disease syndrome (ARDS) (18.0% vs 6.0%). This difference remained statistically significant even with multivariate analysis, that is, after correcting for potential confounding factors.^8^ Furthermore, studies carried out in deceased COVID‐19 individuals revealed pulmonary embolism in large as well as small lung vessels, along with microangiopathy and alveolar capillary microthrombi.^9^, ^10^ At macrovascular level, deep vein thrombosis was found at autopsy in about 60% of the cases, and consequent pulmonary embolism proved to be the direct cause of death in more than a third of the patients with a diagnosis of COVID‐19. Deep vein thrombosis involved the proximal and distal portion of the legs equally.^11^

Even though thrombosis affects peripheral deep vein vessels the most, other regions of the body appear to be involved in patients suffering from COVID‐19 pneumonia. In this respect, the incidence of peripheral arterial thrombosis inducing acute limb ischemia was reported to be significantly increased in northern Italy, one of the worst early epicenters of the pandemic in the world, with worse outcomes than would be expected. Administering antiplatelet or anticoagulant therapy was not as effective as expected. Free‐floating thrombi in the thoracic aorta were described as well in these patients.^12^

With respect to stroke, an incidence rate ranging from 2.5% to 6.4% has been described in COVID‐19 patients.^2^, ^3^, ^13^

Cardiovascular complications are frequent in patients with COVID‐19 and a possible link between SARS‐CoV‐2 and acute myocardial infarction has been suggested. The latter may be the first clinical manifestation of the disease. A majority of patients with COVID‐19 have ST segment elevation on electrocardiogram, thus implying a severe transmural ischemia caused by coronary artery obstruction or rupture of a preexisting atherosclerotic plaque.^14^, ^15^

Finally, systemic thromboembolism is not rare in patients with COVID‐19. A recent study carried out in the United States found that the placenta of mothers affected by COVID‐19 showed thrombi in its large vessels, reduced tissue perfusion and atrophic villi. All babies were born full‐term and tested COVID‐19 negative—suggesting lack of a direct maternal‐fetal transmission; these features were indicative of a systemic hypercoagulable state associated with COVID‐19 infection.^16^ All the cited studies are summarized in Table 1.

**TABLE 1:** COVID‐19‐related thromboembolic events

Author	n	Mean age	Gender	Thromboembolic event(s)	Death
Oxley et al^2^	5	40.4	80% male	Ischemic stroke (100%)	None
Klok et al^3^	184	64	76% male	Venous thromboembolism (27%) Arterial thrombosis (3.7%)	13%
Kollias et al^4^	1,563	52.2	57% male	venous thrombosis (0‐54%)	NR
Akel et al^5^	6	53.1	66.6% male	Pulmonary embolism (100%)	None
Paterson et al^6^	43	62.5	75% male	Ischemic stroke (18.6%) Concomitant pulmonary embolism (9.3%)	12.5%
Middeldorp et al^7^	198	61	66% male	Venous thromboembolism (20%)	19%
Helms et al^8^	150	63	81.3% male	Arterial/venous thrombosis (42.6%) Pulmonary embolism (16.7%)	NR
Ackermann et al^9^	7	74	71.4% male	Pulmonary embolism (100%)	100%
Fox et al^10^	10	63	NR	Pulmonary embolism (100%)	100%
Wichmann et al^11^	12	73	75% male	Venous thromboembolism (58%)	100%
Bellosta et al^12^	20	75	90% male	Acute limb ischaemia (100%)	40%
Stefanini et al^14^	28	68	71.4% male	Intracoronary thrombus (100%)	39.3%
Bangalore et al^15^	18	63	83% male	Intracoronary thrombus (100%)	72%
Mulvey et al^16^	5	32	0% male	Placental thrombosis (100%)	0%

Abbreviation: NR, not reported.

## 3 VIRCHOW'S TRIAD

When looking for a possible pathophysiologic explanation of these observations, one should bear in mind what the eminent German physician Rudolph Ludwig Karl Virchow (1821‐1902) described, about two centuries ago, three broad categories of factors contributing to thrombosis and, hence, thromboembolic risk, namely: endothelial injury, hypercoagulability, and blood stasis.^17^ The definition of “Virchow's triad” was then coined later.

Coronavirus binds to the angiotensin‐converting enzyme 2 (ACE‐2) enzyme, which is widely expressed in multiple organs, including the lung, heart, kidney, and intestine. ACE‐2 receptors are also expressed on the endothelium of blood vessels, thus allowing the virus to enter the bloodstream.^18^ The presence of SARS‐CoV‐2 elements within endothelial cells of different organs was recently demonstrated by Varga et al.^19^ Thus, the first element suggested by Virchow, that is, an endothelial injury, which is caused by SARS‐CoV‐2 virus occurs in multiple organs. SARS‐CoV‐2, once present in the hematic circulation, may invade virtually all organs causing endothelial injury, systemic vasculitis, myocarditis, encephalitis, and multi‐organ failure. The inflamed and injured blood vessels facilitate the recruitment of leukocytes, macrophages, and mast cells, which have important roles in the immunity. The more aggressive the disease, the more massive the recruitment. An aggressive viral infection induces apoptosis of lymphocytes and consequent lymphopenia as well.^20^ The protective immune system leads to the release of molecules such as C‐reactive protein, fibrinogen, ferritin, D‐dimer, and the pro‐inflammatory cytokines, including interleukin‐6 (IL‐6). A progressive dysregulated coagulative response to SARS‐CoV‐2 viral infection with the synthesis of IL‐6 and other inflammatory mediators (the so‐called cytokine storm) contribute in turn to activate the complement system, clotting cascade, thus causing a state of hypercoagulability.^3^

As to the factors contributing to hypercoagulability, a recent intriguing theory involves the role of neutrophil extracellular traps (NETs)—which are extracellular webs made up of chromatin, microbicidal proteins, and oxidant enzymes released by neutrophils to contain infections. Research studies were mostly focused on macrophages and endothelial cells in COVID‐19 pathophysiology, with little attention paid to neutrophils. NETs trigger thrombosis in arteries and veins through a complex interplay with contact and intrinsic pathways of coagulation. The platelet inhibitor dipyridamole seems to inhibit NETs formation. Since NETs proved to be over‐expressed in COVID‐19 patients, NETs formation may well be a new therapeutic target in severe cases of COVID‐19.^21^ On balance, COVID‐19‐associated coagulopathy has its own unique and distinctive features, which involve the immune system. This is exemplified by derangements in laboratory tests as well, such as usual increase in D‐dimer and fibrinogen levels and initially minimal abnormalities in prothrombin time and platelet count.^22^

**FIGURE 1:**
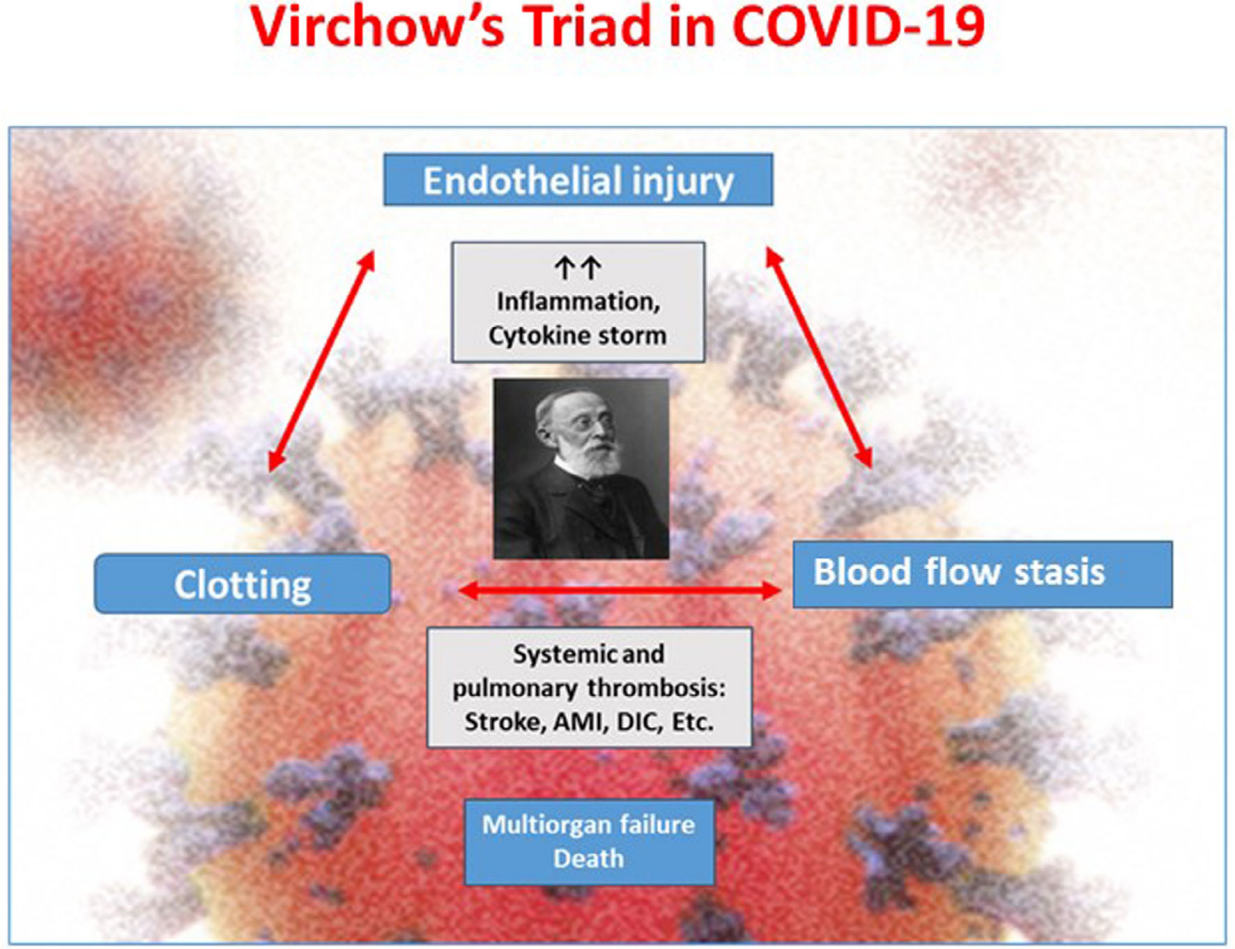
Virchow's Triad and COVID‐19. Three cardinal component of Virchow's Triad—endothelial injury, clotting and blood flow stasis—are often observed in COVID‐19 resulting in systemic and venous thrombosis and their manifestations—such as stroke, acute myocardial infarction (AMI), primary thrombosis, and disseminated intravascular coagulation (DIC)

Finally, the third component of Virchow's triad, that is, blood stasis, comes from prolonged bedrest and immobilization in intensive care unit, strict isolation, and limited physiotherapy. These concepts are shown in the Figure 1.

One may certainly argue that Virchow's triad explains venous thromboembolic events (venous thrombosis, pulmonary embolism) better than the arterial (ischemic stroke, myocardial infarction, acute limb ischaemia), but this is not completely true. In fact, the majority of arterial thromboembolic events share the same origin, that is, the formation of a clot over an underlying atherosclerotic plaque. In most cases, the thrombus overlies a ruptured plaque or an intact plaque with superficial endothelial erosion.^23^ In this respect, the first element of Virchow's triad is still in place, since SARS‐CoV‐2 can infect arterial endothelial cells directly, thus causing endothelial injury. For example, viral particles have been detected in endothelial cells of injured artery in a patient with past medical history of renal transplantation who passed away from COVID‐19‐induced multi‐organ failure.^19^ As soon as arterial wall is damaged, the innate immune system is activated. In particular, macrophages begin to secrete collagenases which in turn degrade collagen, a major constituent of the fibrous cap on atherosclerotic plaques, thus causing plaque rupture. Activated macrophages also release tissue factor, a potent procoagulant factor which triggers clot formation when the plaque is broken.^24^ Patients with COVID‐19 often have comorbidities leading to atherosclerosis, like hypertension and diabetes, and arterial plaques constitute a major risk factor for platelet adhesion leading to arterial thrombosis. SARS‐CoV‐2 infection is associated with platelets hyperreactivity in terms of increased aggregation, which can partially be attributed to increased mitogen‐activated protein kinase activation and thromboxane generation^25^ (hypercoagulability). While venous thrombi mainly consist of fibrin and red blood cells, and less by platelets, the latter are essential in arterial thrombus formation in an attempt to repair the damaged endothelium, which in turn is the main stimulus triggering platelets activation and arterial hypercoagulability.^26^ As to the third element of Virchow's triad (blood stasis), there is no doubt that, when an arterial lumen is occluded partially or in entirety by an atherosclerotic plaque, blood flow at that levels slows down or is completely shut down.^27^

## 4 THERAPEUTIC OPTIONS: DO THEY HAVE VIRCHOW'S TRIAD ELEMENTS AS A TARGET?

Some of the drugs proposed for COVID‐19, and others currently undergoing evaluation, may have a beneficial effect on the COVID‐19–related coagulopathy, having one or more of the Virchow's triad elements as a target.

At the present time, among the benchmarks of COVID‐19 therapy are antiaggregant/anticoagulants as well as antivirals/immunomodulating agents.

Aspirin, unfractionated heparin, low molecular weight heparin, old and new oral anticoagulants belong to the first category. Most of them have hypercoagulability as a therapeutic target. Although aspirin is usually not included in the treatment of COVID‐19, it has the triple effects of inhibiting virus replication (by inhibiting prostaglandin E2 in macrophages and upregulating type I interferon production), antiaggregant (blood thinning is by irreversibly blocking the formation of thromboxane A_2_ in platelets, thus inducing an inhibitory effect on their aggregation), and anti‐inflammatory (by inhibiting cyclooxygenases‐oxidase). Based on these premises, a trial (PEAC [Protective Effect of Aspirin on COVID‐19 patients]) to test the effect of aspirin in the COVID‐19 scenario is ongoing.^28^ Unfractionated heparin, after binding antithrombin III, is able to inactivate thrombin (factor IIa), factor X, and other proteases involved in coagulation pathway. Conversely, low molecular weight heparins are capable of inhibiting clotting factor X, but not thrombin. At this time, it is difficult to say as to which is better treatment between unfractionated heparin and low molecular weight heparin. Unfractionated heparin may be preferred when there is renal insufficiency or need for reversibility for an urgent intervention.^29^ Both old and new oral anticoagulants display significant interference with concomitant antiviral treatment which is administered to COVID‐19 patients. So, an individualized patient‐based (“tailored”) approach is recommended, aimed at balancing the risk/benefit ratio of the various antithrombotic strategies, taking into account his/her underlying hypercoagulable state.^30^

As to the second category, immunomodulatory therapy administered in subjects with severe COVID‐19 is likely to share other effects beyond the antiviral therapy. In particular, IL‐6 blockers like tocilizumab and sarilium could prevent the “cytokine storm” in severe forms of COVID‐19, and secondarily inhibit hypercoagulability. Other drugs acting against other types of interleukins might have a similar though weaker effect.^31^

Other drugs may potentially have a component of Virchow's triad as a target. For example, old antimalarial agents like chloroquine and hydroxychloroquine may prevent endothelial injury (endothelialitis). Their proposed mechanism of action relates to their ability to prevent membrane fusion, disrupting SARS‐CoV‐2 ability to enter cells and begin replication.^32^ Recently, concerns have been raised about their safety in COVID‐19 setting, with controversial evidence.^33^

Investigations—in terms of randomized trials—regarding other medications potentially capable of influencing platelet‐endothelial cell interactions (hypercoagulability) and preventing endothelial cell damage (endothelial injury) in COVID‐19 hypercoagulable state are urgently needed.

## 5 CONCLUSION

On balance, COVID‐19, the pandemic disease caused by SARS‐CoV‐2, is increasingly being recognized as a systemic thrombotic illness with systemic activation of the coagulation cascade and secondary DIC as well. It causes multi‐organ involvement, which dramatically worsen patients' outcome.

Hypercoagulability is probably the reason why many investigators worldwide are suggesting prophylactic use of pharmacological agents against thrombosis—at a very high‐dose—in COVID‐19 patients especially those who are very sick, even in the absence of evidence from randomized clinical trials.^34^

We suggest that the three components of the classic Virchow's triad are the basis of thrombosis in COVID‐19 patients.

## CONFLICT OF INTEREST

The authors declare no potential conflict of interests.

## Data Availability

All the data included in this manuscript were available to all authors.
